# Effects of *GADL1* overexpression on cell migration and the associated morphological changes

**DOI:** 10.1038/s41598-019-41689-x

**Published:** 2019-03-28

**Authors:** Tai-Na Wu, Chih-Ken Chen, I-Chao Liu, Lawrence Shih-Hsin Wu, Andrew Tai-Ann Cheng

**Affiliations:** 1 Institute of Biomedical Sciences, Academia Sinica, Taipei, Taiwan; 2 School of Medicine, Chang-Gung University and Chang-Gung Memorial Hospital, Keelung, Taiwan; 3 School of Medicine, Fu Jen Catholic University and Fu Jen Catholic University Hospital, New Taipei, Taiwan; 4 Graduate Institute of Biomedical Sciences, China Medical University, Taichung, Taiwan

## Abstract

Lithium has been used for maintenance treatment of bipolar disorder, but drug response varies among patients. Single-nucleotide polymorphisms in glutamate decarboxylase–like protein 1 (*GADL1*) are found to be associated with lithium response in Han Chinese bipolar patients. In this study, we assessed GADL1 function using a neuroblastoma cell line that stably overexpressed GADL1. Genes encoding factors involved in cell migration, such as *FN1*, *ITGA2*, *ITGAV* and *CCL2*, were downregulated in *GADL1*-overexpressing cells. *GADL1* overexpression indeed suppressed cell migration. Cell migration speed and perimeter length exhibited similar trends, both of which were decreased under *GADL1* overexpression or lithium treatment but increased upon stimulation with CCL2. Secreted GADL1 or its enzyme product, taurine, in the conditioned medium might exert only mild effects on the observed changes. Compared with SH-SY5Y cells, *GADL1*-overexpressing cells were much more sensitive to CCL2 treatment but less sensitive to lithium, indicating that the level of *GADL1* expression can affect cell sensitivity to lithium or  CCL2 treatment. Together, these results suggest that cell migration and related morphological changes might provide good indicators of the sensitivity toward lithium treatment, and the *GADL1* stable overexpression cell line might serve as a useful platform to screen novel therapeutics for bipolar disorder.

## Introduction

Bipolar disorder is a disabling mental illness that is characterized by episodes of both elevated or irritable mood (mania) and depression^1,2^. Currently, lithium is the first-line mood stabilizer for maintenance treatment of bipolar disorder and reduces the risk of both relapse and suicide^3–5^. However, only 30% of patients who are treated with lithium have an excellent response with complete remission of symptoms observed in patients of European descent^6,7^. Our previous genome-wide association study demonstrated that single-nucleotide polymorphisms in the gene encoding glutamate decarboxylase–like protein 1 (*GADL1*) are associated with lithium response in bipolar patients of Han Chinese descent^8^. Although our findings have not yet been replicated with clinical samples from different populations^9,10^, to define the role of GADL1 in lithium response in neuropsychiatric disorders requires further investigation.

GADL1 has aspartate 1-decarboxylase and cysteine sulfinic acid decarboxylase activities, can therefore catalyze decarboxylation of aspartate, cysteine sulfinic acid, and cysteic acid to produce β-alanine, hypotaurine, and the sulfur-containing amino acid, taurine (2-amino-ethanesulfonic acid)^11^. Taurine is abundant in certain mammalian tissues, such as brain, spinal cord, retina, heart and muscle, and has many physiological functions^12^. For example, taurine helps maintain osmotic pressure and preserve the structural integrity of membranes^13,14^. In the nervous system, taurine may act as a trophic factor^15^ or neuromodulator^16,17^.

In humans, *GADL1* is expressed in neurons. In 3-week-old mice, *Gadl1* expression is higher in the olfactory bulb than in the liver or kidney^18^. In the adult mammalian forebrain, the olfactory bulb is an active zone for neuron regeneration. Stem cells of the subventricular zone give rise to neuroblasts that migrate tangentially along the rostral migratory stream until they reach the olfactory bulb, where they then migrate radially to complete their differentiation into neurons^19–21^.

Fibronectins reside in the extracellular matrix and are involved in cell adhesion and migration processes as well as the maintenance of cell shape. They are one of the ligands that bind integrins, which are transmembrane receptors that couple the extracellular matrix to the cytoskeleton to regulate cell migration^22,23^. The chemokine (C-C motif) ligand 2 (CCL2) also regulates neuron migration^24,25^. Treatment of neurons in culture with CCL2 leads to a significant, dose-dependent increase in the number of migrating neurons and the average distance they travel^25^. Neurons that have undergone *in vitro* transdifferentiation from bipolar patient skin cells exhibit significantly different cell-adhesion phenotypes between lithium responders and nonresponders, indicating that cell adhesion is associated with clinical response to lithium treatment^26^. To help understand GADL1 function, *GADL1* was stably overexpressed in the human neuroblastoma cell line, SH-SY5Y. We assessed the impact of *GADL1* overexpression or of treatment with lithium or CCL2 on cell migration and related morphological changes.

## Results

### *GADL1* overexpression downregulates genes involved in cell migration

**Figure 1:**
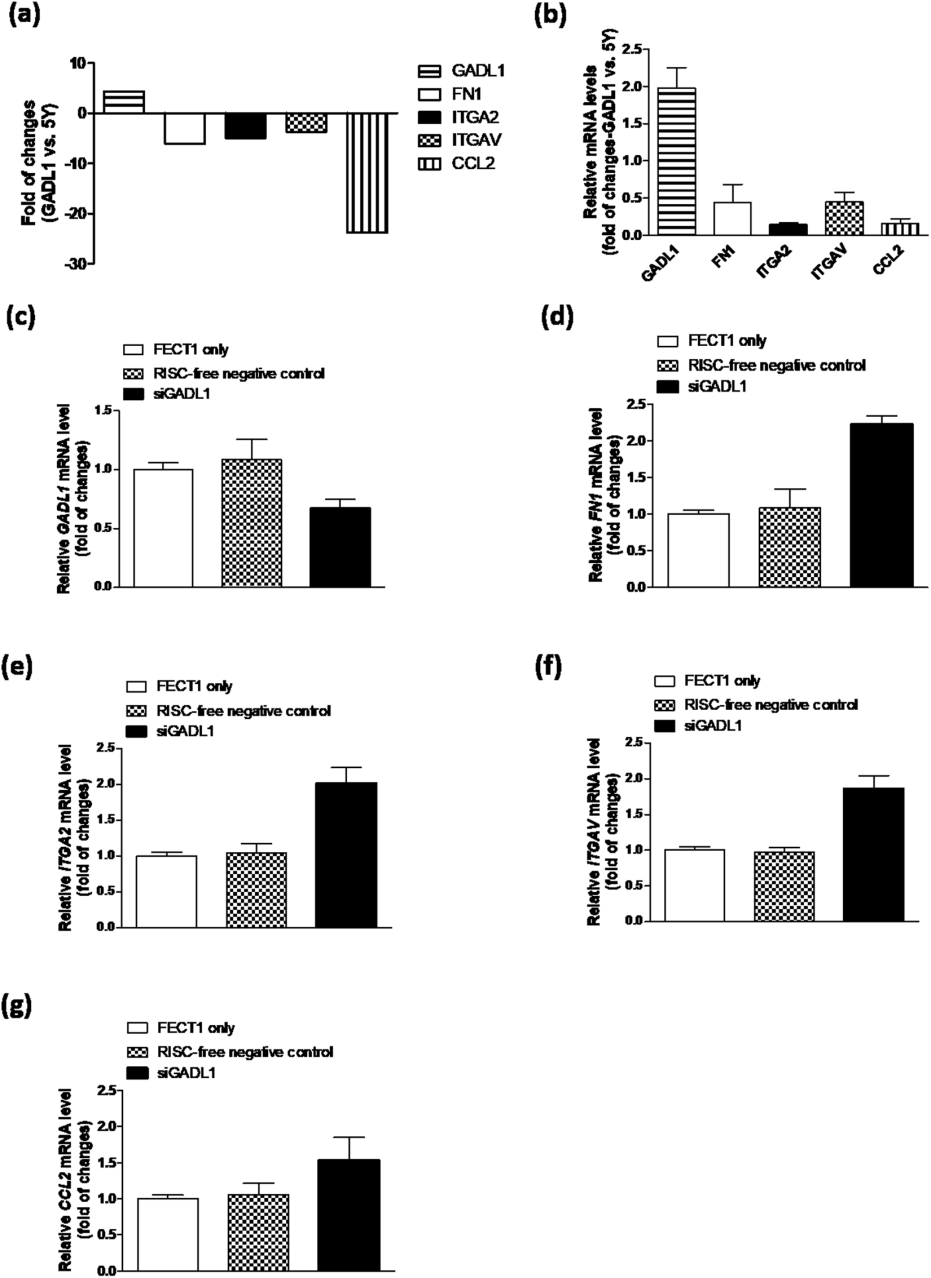
Genes downregulated upon *GADL1* overexpression. (**a**) RNA expression array analyses were used to determine the levels of *GADL1*, *FN1*, *ITGA2*, *ITGAV* and *CCL2* mRNAs in the *GADL1*-overexpressing cells (GADL1) relative to the parental cell line, SH-SY5Y (5Y). (**b**) Total RNA from cells was reverse transcribed into cDNA and subjected to RT-qPCR analysis for *GADL1*, *FN1*, *ITGA2*, *ITGAV* and *CCL2*. Data were normalized to that for *ACTB* in each sample, and the fold-change value for each gene is shown for *GADL1*-overexpressing cells relative to SH-SY5Y cells. RNA samples for expression microarray analysis and RT-qPCR validation were prepared independently. (**c–g**) *GADL1*-overexpressing cells were transfected with RISC-free negative control siRNA or siRNA targeting GADL1 (siGADL1) at 0.1 μM using DharmaFECT1 (FECT1) transfection reagent. Total RNA from cells was reverse transcribed into cDNA and subjected to RT-qPCR analysis for (**c**) *GADL1*, (**d**) *FN1*, (**e**) *ITGA2*, (**f**) *ITGAV* and (**g**) *CCL2*. The fold-change value for each gene was normalized to *ACTB* expression. (**b–g**) Data were combined from two independent experiments.

Total RNA extracted from cells was analyzed with a RNA expression array, which revealed that 118 genes were upregulated (≥2-fold increase) and 399 genes were downregulated (≤–2-fold decrease) upon *GADL1* overexpression. *GADL1* was indeed overexpressed in the stable clone as compared with the parental line SH-SY5Y, whereas fibronectin 1 (*FN1*), integrin subunit alpha 2 (*ITGA2*), integrin subunit alpha V (*ITGAV*), and *CCL2* were downregulated (Fig. 1a). These data were validated with real-time quantitative PCR (RT-qPCR), revealing that *GADL1* was upregulated (1.98-fold increase) and that the other four genes (*FN1*, 0.44-fold decrease; *ITGA2*, 0.14-fold decrease; *ITGAV*, 0.45-fold decrease; *CCL2*, 0.15-fold decrease) were downregulated in the stable clone (Fig. 1b).

To demonstrate a causative relationship between *GADL1* overexpression and the cellular phenotypes, we further reduced *GADL1* expression in the *GADL1*-overexpressing cell line using small interfering RNA (siRNA) knockdown. The RNA expression changes of *GADL1*, *FN1*, *ITGA2*, *ITGAV*, and *CCL2* after *GADL1* knockdown (siGADL1) in the *GADL1*-overexpressing cell line were examined using RT-qPCR analysis, showing that *GADL1* was knocked down to 67.2% relative to RISC-free control siRNA (Fig. 1c). *FN1* (2.22-fold increase, Fig. 1d), *ITGA2* (2.02-fold increase, Fig. 1e), *ITGAV* (1.87-fold increase, Fig. 1f), and *CCL2* (1.53-fold increase, Fig. 1g) were upregulated after siGADL1 treatment.

### Effects of *GADL1* overexpression on cell number, migration, and morphology

**Figure 2:**
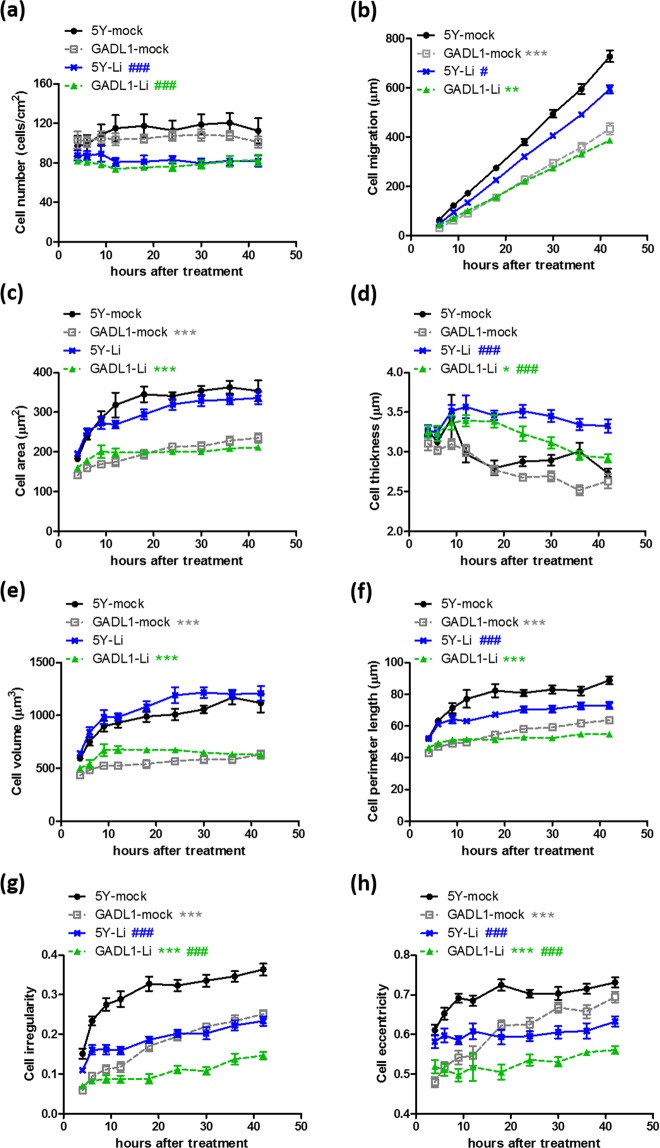
Lithium effects on cell number, migration and morphology. (**a**) Cell number, (**b**) cell migration distance, and morphological changes of cells including (**c**) cell area, (**d**) thickness, (**e**) volume, (**f**) perimeter length, (**g**) irregularity, and (**h**) eccentricity were measured using real-time, three-dimensional holographic imaging. At 4–5 h after seeding of SH-SY5Y (5Y) cells or *GADL1*-overexpressing cells (GADL1), 20 mM lithium was added, and images were acquired at 20-min intervals for 48 h. Data were mean ± s.e.m. values from one experiment and were representative of three independent experiments. Repeated measure ANOVA with Tukey’s multiple comparison test was used to compare the differences between SH-SY5Y and *GADL1*-overexpressing cells (^*^p < 0.05; ^**^p < 0.01; ^***^p < 0.001) or between mock and lithium treatment (^#^p < 0.05; ^###^p < 0.001).

Next, cell migration was compared between *GADL1*-overexpressing and SH-SY5Y cells using label-free, real-time, holographic imaging for 48 h (Fig. 2). Cell counts and morphology were also monitored at the same time. Cell number (Fig. 2a) and thickness (Fig. 2d) did not differ significantly between the *GADL1*-overexpressing cells and SH-SY5Y cells. Moreover, *GADL1* overexpression significantly decreased cell migration (Fig. 2b), area (Fig. 2c), volume (Fig. 2e), and perimeter length (Fig. 2f).

Two parameters were also used to assess changes in cell shape: irregularity and eccentricity. Irregularity, calculated as 1–4π(area)/(perimeter length)^2^, is how much the circumference of the cell deviates from the circumference of a perfect circle. A value of 0 means the cell is circular, whereas a higher value means a more irregular (and hence longer) perimeter. Eccentricity, referring to the elongation of a cell, is calculated as the square root of 1 − w^2^/h^2^ where h and w are the height and width of the minimum rectangle, w ≤ h. A value of 0 means that cells are essentially square, whereas a higher value means that cells are more elongated, i.e., rectangular. *GADL1* overexpression significantly decreased both cell irregularity (Fig. 2g) and eccentricity (Fig. 2h), which could be observed by phase contrast microscopy as shown in Figs S1 and S2.

### Effects of lithium on cell number, migration, and morphology

The single-nucleotide polymorphisms in *GADL1* have been found to be associated with lithium response in bipolar patients of Han Chinese descent^8^. As shown in Fig. 1, *GADL1* overexpression downregulated certain genes, including *FN1*, *ITGA2*, and *ITGAV*, involved in cell adhesion and migration^22^. Thus, we hypothesized that lithium could affect cell adhesion/migration, which might be affected by the cellular level of GADL1. To test this idea, we monitored cell migration, cell counts, and morphology using label-free, real-time, holographic imaging of *GADL1*-overexpressing and SH-SY5Y cells in the presence or absence of 20 mM lithium chloride for 48 h (Fig. 2). Treatment with lithium at 1 mM did not have powerful effects on cellular phenotypes for both cells, except on cell area and perimeter length (Fig. S3).

For both SH-SY5Y and *GADL1-*overexpressing cells, lithium exposure significantly decreased cell number (Fig. 2a), irregularity (Fig. 2g), and eccentricity (Fig. 2h), but lithium had the effect of increasing cell thickness (Fig. 2d). Lithium had only mild effects on cell area (Fig. 2c) and cell volume (Fig. 2e). Lithium-induced morphological changes on SH-SY5Y and *GADL1-*overexpressing cells could be also observed by phase contrast microscopy as shown in Figs S1 and S2, respectively. Treatment with lithium significantly decreased cell migration (Fig. 2b) and perimeter length (Fig. 2f) only in SH-SY5Y cells.

The cell migration curves were subjected to linear regression analysis. The slope (migration speed, μm/h) derived from each curve was as follows: 5Y-mock, 18.1 ± 0.41; GADL1-mock, 11.2 ± 0.35; 5Y-Li, 15.0 ± 0.27; GADL1-Li, 9.6 ± 0.18. Lithium treatment decreased cell migration speed in both SH-SY5Y and *GADL1-*overexpressing cells, and the speed decrease was more evident for SH-SY5Y cells (∆ = −3.1) than *GADL1*-overexpressing cells (∆ = −1.6). Lithium induced significant speed decreases for SH-SY5Y cells, but not for *GADL1*-overexpressing cells, as analyzed by repeated measure ANOVA to calculate changes of the entire curve (Fig. 2b). Regarding cell migration speed, *GADL1*-overexpressing cells were less sensitive to lithium than SH-SY5Y cells. After 42 h of lithium exposure, cell perimeter length decreased more obviously for SH-SY5Y cells (−11.9 μm) than *GADL1*-overexpressing cells (−8.7 μm) (Fig. 2f). These findings revealed that *GADL1* overexpression decreased the sensitivity of the cells to lithium—especially reflected by its effects on cell migration and perimeter length.

### Effects of CCL2 treatment on cell number, migration, and morphology

**Figure 3:**
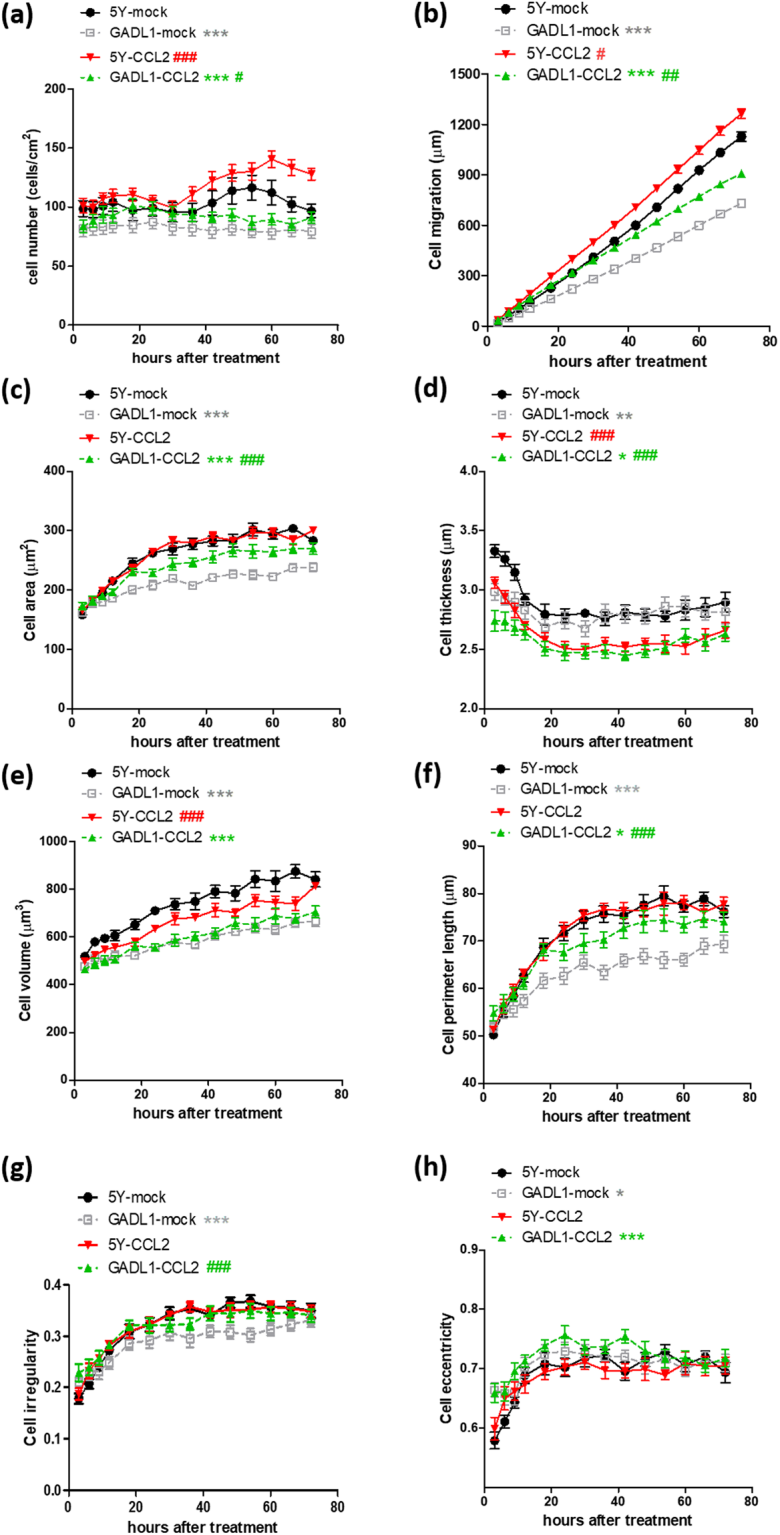
CCL2 effects on cell number, migration and morphology. The (**a**) cell number, (**b**) cell migration distance, and morphological changes of cells including (**c**) cell area, (**d**) thickness, (**e**) volume, (**f**) perimeter length, (**g**) irregularity, and (**h**) eccentricity were measured using real-time, three-dimensional holographic imaging. At 4–5 h after seeding of SH-SY5Y (5Y) cells or *GADL1*-overexpressing cells (GADL1), 50 ng/ml CCL2 was added, and images were acquired at 20-min intervals for 72 h. Data were mean ± s.e.m. values from one experiment and were representative of four independent experiments. Repeated measure ANOVA with Tukey’s multiple comparison test was used to compare the differences between SH-SY5Y and *GADL1*-overexpressing cells (^*^p < 0.05; ^**^p < 0.01; ^***^p < 0.001) or between mock and CCL2 treatment (^#^p < 0.05; ^##^p < 0.01; ^###^p < 0.001).

CCL2 can regulate neuronal migration *in vi*tro^24,25^, and we found that *GADL1* overexpression downregulated *CCL2* expression (Fig. 1). Thus, we assessed the effects of CCL2 alone or in concert with *GADL1* overexpression on cell migration, cell counts, and morphology using label-free, real-time, holographic imaging of *GADL1*-overexpressing and SH-SY5Y cells in the presence or absence of 50 ng/ml CCL2 for 72 h (Fig. 3). Treatment with CCL2 at 25 ng/ml did not significantly affect the examined parameters (data not shown).

For both SH-SY5Y and *GADL1*-overexpressing cells, stimulation with 50 ng/ml CCL2 significantly decreased cell thickness (Fig. 3d) but increased cell number (Fig. 3a) and migration (Fig. 3b). Cell eccentricity (Fig. 3h) did not change for either cell type in response to CCL2. In comparison, CCL2 exposure increased cell area (Fig. 3c), perimeter length (Fig. 3f), and irregularity (Fig. 3g) only in *GADL1*-overexpressing cells. Treatment with CCL2 decreased cell volume (Fig. 3e) only in SH-SY5Y cells.

The cell migration curves were subjected to linear regression analysis. The slope (migration speed, μm/h) derived from each curve was as follows: 5Y-mock, 16.1 ± 0.18; GADL1-mock, 10.3 ± 0.18; 5Y-CCL2, 17.8 ± 0.20; GADL1-CCL2, 12.7 ± 0.16. Therefore, for both SH-SY5Y and *GADL1*-overexpressing cells, CCL2 treatment increased cell migration speed, and this increase was more evident for *GADL1*-overexpressing cells (∆ = +2.4) than SH-SY5Y cells (∆ = +1.7). Regarding CCL2-induced speed increases, *GADL1*-overexpressing cells (p < 0.01) were more sensitive to CCL2 treatment than SH-SY5Y cells (p < 0.05), as analyzed by repeated measure ANOVA to calculate changes of the entire curve (Fig. 3b). After 42 h of CCL2 treatment, the increase in cell perimeter length was more obvious for *GADL1*-overexpressing cells (+6.9 μm) than SH-SY5Y cells (+1.1 μm) (Fig. 3f). These findings revealed that *GADL1* overexpression increased the cell sensitivity to CCL2—especially reflected by its effects on cell perimeter length.

### The effects of *GADL1* overexpression on cell migration and morphology as well as the sensitivity toward lithium are cell autonomous

**Figure 4:**
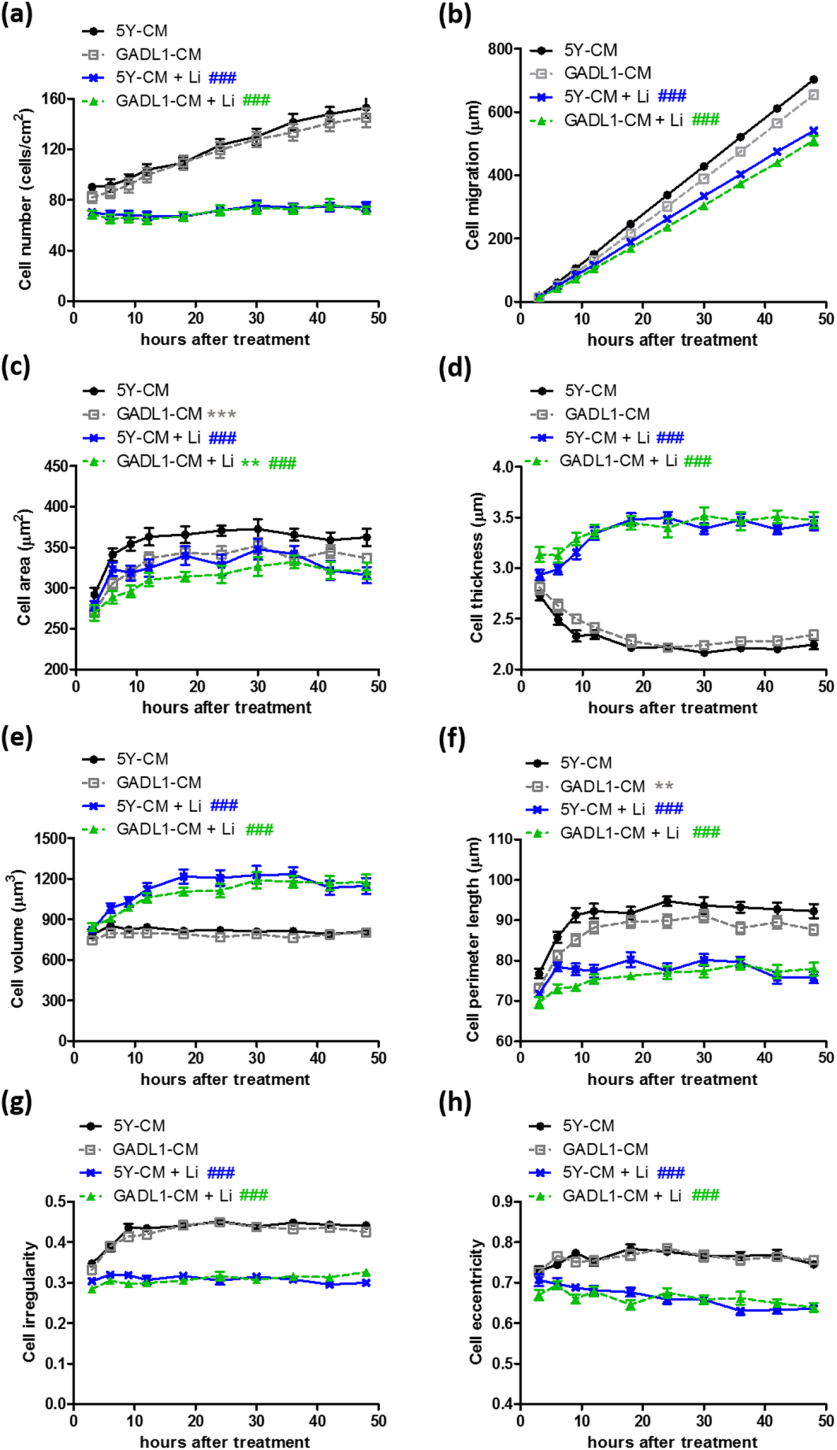
The effects of *GADL1* overexpression on cell migration and morphology as well as the sensitivity toward lithium are cell autonomous. (**a**) Cell number, (**b**) cell migration distance, and morphological changes of cells including (**c**) cell area, (**d**) thickness, (**e**) volume, (**f**) perimeter length, (**g**) irregularity, and (**h**) eccentricity were measured using real-time, three-dimensional holographic imaging. SH-SY5Y cells were cultured in the conditioned medium (CM) from SH-SY5Y cells (5Y-CM) or from *GADL1*-overexpressing cells (GADL1-CM). At 4–5 h after cell seeding, 20 mM lithium was added, and images were acquired at 20-min intervals for 48 h. Data were mean ± s.e.m. values from one experiment and were representative of four independent experiments. Repeated measure ANOVA with Tukey’s multiple comparison test was used to compare differences between 5Y-CM and GADL1-CM (^**^p < 0.01; ^***^p < 0.001) or between mock and lithium treatment (^###^p < 0.001).

Because GADL1 is an enzyme^11^, we speculated whether the effects of GADL1 are cell autonomous or non-autonomous. To address this question, conditioned medium (CM) was collected from 2- to 3-day cultures of *GADL1*-overexpressing (GADL1-CM) or SH-SY5Y (5Y-CM) cells. Figure 4 shows that neither CM substantially affected SH-SY5Y cells in terms of cell number (Fig. 4a), migration (Fig. 4b), thickness (Fig. 4d), volume (Fig. 4e), irregularity (Fig. 4g), and eccentricity (Fig. 4h). As compared with 5Y-CM, SH-SY5Y cells cultured in GADL1-CM exhibited decreased cell area (Fig. 4c) and perimeter length (Fig. 4f). However, the decrease in cell area and perimeter length induced by GADL1-CM was much smaller than that induced by *GADL1*-overexpressing cells, as shown in the comparisons in Fig. 2c,f, respectively. These results suggested that the *GADL1* overexpression–induced decrease in cell area and perimeter length was mainly dependent on the ‘intracellular form’ of GADL1 rather than the ‘secreted form’.

Regardless of whether SH-SY5Y cells were cultured in 5Y-CM or GADL1-CM, lithium exposure significantly decreased cell number (Fig. 4a), migration (Fig. 4b), area (Fig. 4c), perimeter length (Fig. 4f), irregularity (Fig. 4g), and eccentricity (Fig. 4h), but lithium increased cell thickness (Fig. 4d) and volume (Fig. 4e). The cell migration curves were subjected to linear regression analysis. The slope (migration speed, μm/h) derived from each curve was as follows: 5Y-CM, 15.3 ± 0.16; GADL1-CM, 14.3 ± 0.18; 5Y-CM + Li, 11.8 ± 0.16; GADL1-CM + Li, 11.1 ± 0.17. For SH-SY5Y cells cultured in 5Y-CM or GADL1-CM, lithium treatment significantly decreased cell migration speed, but the magnitude of the speed decrease was similar between 5Y-CM (∆ = −3.5) and GADL1-CM (∆ = −3.2). In comparison, the lithium-induced decrease in migration speed between SH-SY5Y cells (∆ = −3.1) and *GADL1*-overexpressing cells (∆ = −1.6) was more obvious (Fig. 2b). Taken together, these results indicated that the *GADL1* overexpression–induced differential sensitivity toward lithium was cell autonomous and depended mainly on the intracellular form of GADL1.

### The effects of *GADL1* overexpression on the sensitivity to CCL2 are cell autonomous

To understand if the differential sensitivity to CCL2 was mediated by the intracellular or secreted form of GADL1, CM was collected from 2- to 3-day cultures of *GADL1*-overexpressing (GADL1-CM) or SH-SY5Y cells (5Y-CM). Then, SH-SY5Y cells were cultured in the GADL1-CM or 5Y-CM in the presence or absence of CCL2 (50 ng/ml).

**Figure 5:**
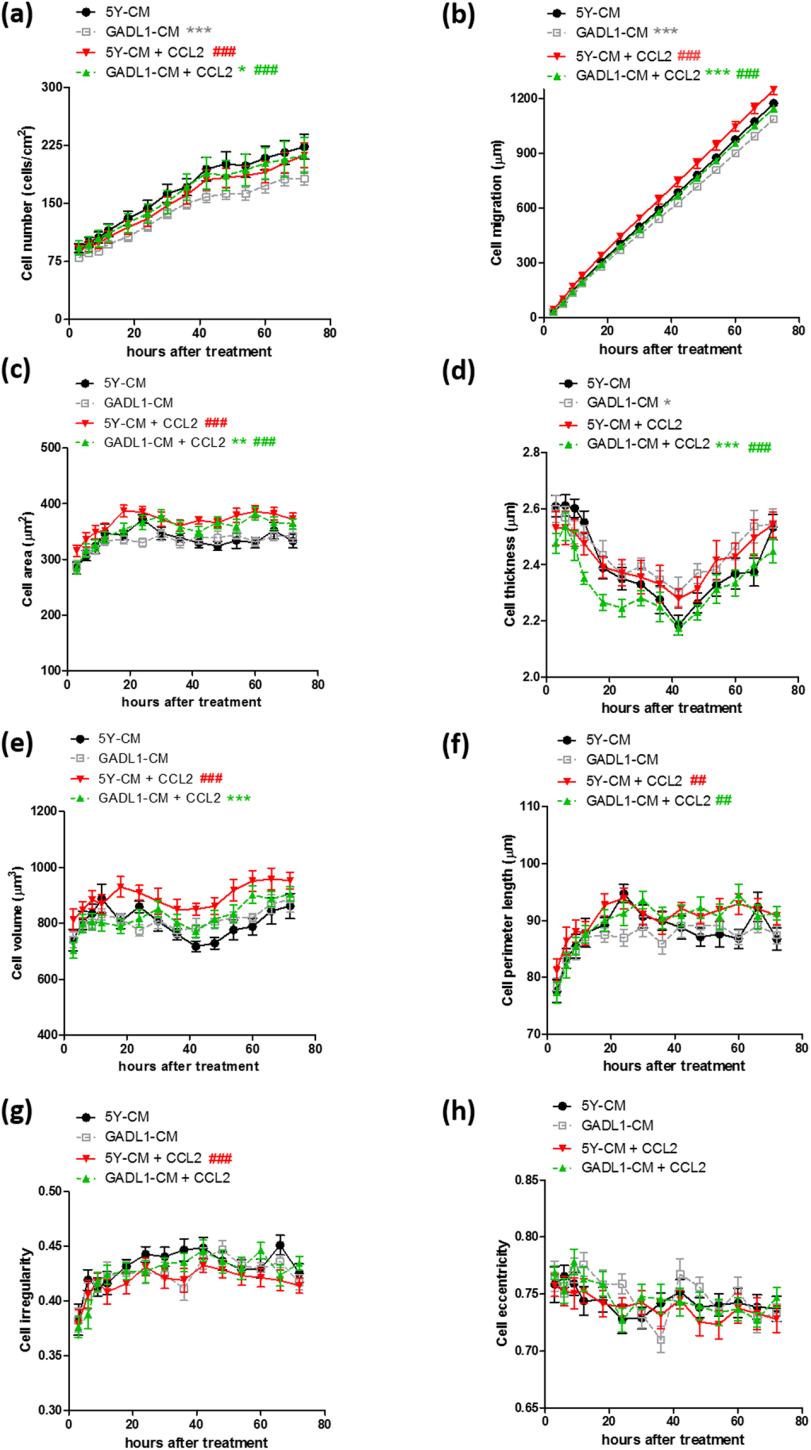
The effects of *GADL1* overexpression on the sensitivity to CCL2 are cell autonomous. (**a**) Cell number, (**b**) cell migration distance, and morphological changes of cells including (**c**) cell area, (**d**) thickness, (**e**) volume, (**f**) perimeter length, (**g**) irregularity, and (**h**) eccentricity were measured using real-time, three-dimensional holographic imaging. SH-SY5Y cells were cultured in the conditioned medium (CM) from SH-SY5Y cells (5Y-CM) or from *GADL1*-overexpressing cells (GADL1-CM). At 4–5 h after cell seeding, 50 ng/ml CCL2 was added, and images were acquired at 20-min intervals for 72 h. Data were mean ± s.e.m. values from one experiment and were representative of four independent experiments. Repeated measure ANOVA with Tukey’s multiple comparison test was used to compare differences between 5Y-CM and GADL1-CM (^*^p < 0.05; ^**^p < 0.01; ^***^p < 0.001) or between mock and CCL2 treatment (^##^p < 0.01; ^###^p < 0.001).

Treatment with CCL2 significantly increased SH-SY5Y cell volume (Fig. 5e) but decreased irregularity (Fig. 5g) only in 5Y-CM. In comparison, CCL2 treatment significantly decreased SH-SY5Y cell thickness (Fig. 5d) only in GADL1-CM. CCL2 stimulation had different effects on cell number (Fig. 5a) when SH-SY5Y cells were cultured in 5Y-CM vs. GADL1-CM. Regardless of whether SH-SY5Y cells were cultured in 5Y-CM or GADL1-CM, CCL2 exposure did not substantially affect SH-SY5Y cell eccentricity (Fig. 5h), but CCL2 addition did increase cell migration (Fig. 5b), area (Fig. 5c), and perimeter length (Fig. 5f). The cell migration curves were subjected to linear regression analysis. The slope (migration speed, μm/h) derived from each curve was as follows: 5Y-CM, 16.4 ± 0.11; GADL1-CM, 15.1 ± 0.09; 5Y-CM + CCL2, 17.2 ± 0.23; GADL1-CM + CCL2, 16.0 ± 0.13. For SH-SY5Y cells cultured in either 5Y-CM or GADL1-CM, treatment with CCL2 significantly increased cell migration speed, but the increase was similar between 5Y-CM (∆ = + 0.8) and GADL1-CM (∆ = + 0.9). In comparison, the CCL2-induced increase in migration speed between SH-SY5Y cells (∆ = +1.7) and *GADL1*-overexpressing cells (∆ = +2.4) was more pronounced (Fig. 3b). Taken together, these results suggested that the *GADL1* overexpression–induced differential sensitivity to CCL2 was cell autonomous and depended mainly on the intracellular form of GADL1.

### Effects of *GADL1* overexpression or treatment with lithium or CCL2 on cell migration speed and perimeter length as assessed in independent experiments

**Figure 6:**
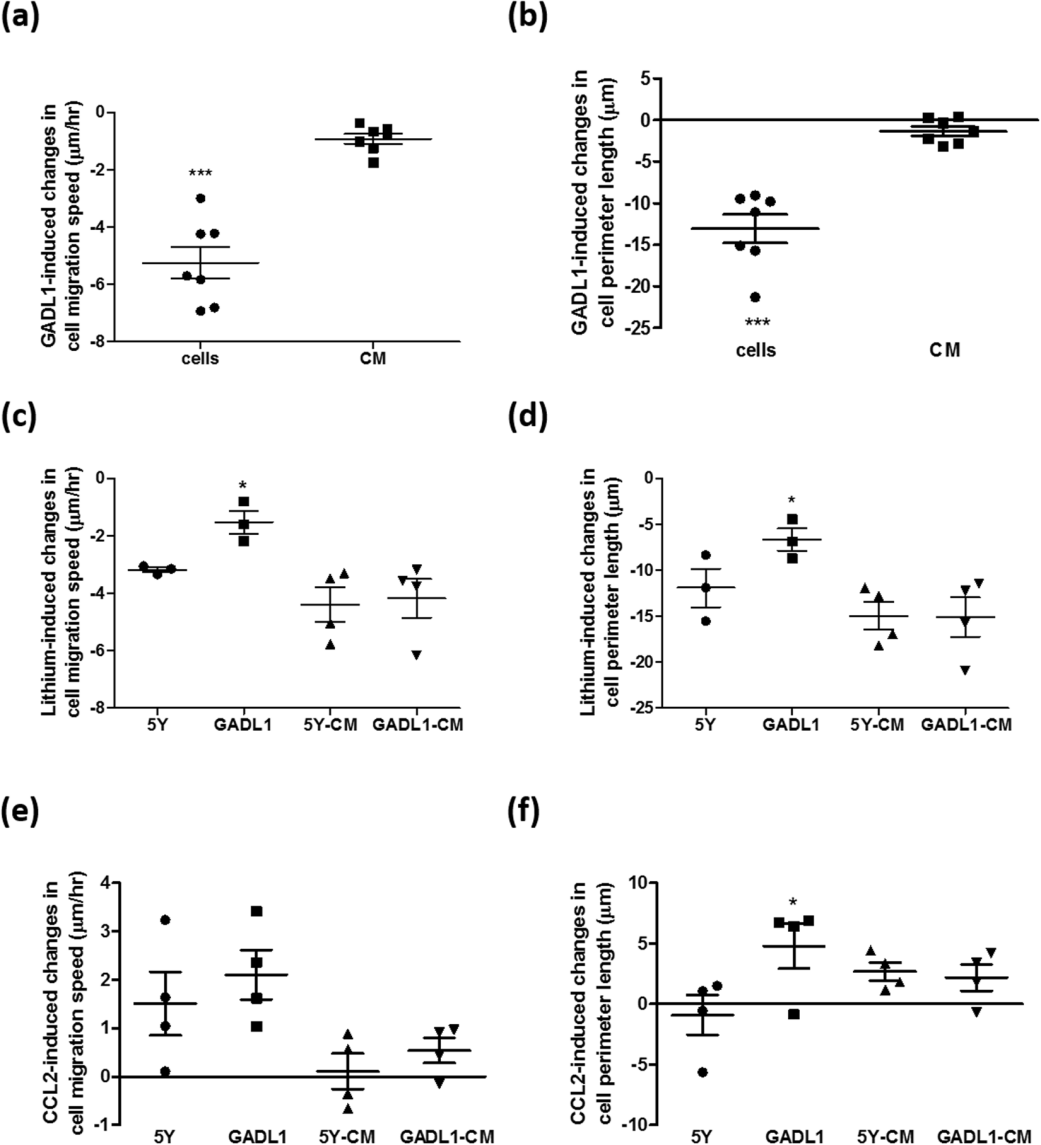
Effects of *GADL1* overexpression or lithium or CCL2 treatments on cell migration speed and perimeter length from independent experiments. Data from different batches of experiments were statistically analyzed to confirm *GADL1* overexpression and the effects of treatment with lithium (20 mM) or CCL2 (50 ng/ml) on cell migration speed (a, c, e, respectively), and perimeter length recorded at 42 h (b, d, f, respectively). The Student’s t test was used to compare the differences between SH-SY5Y (5Y) cells and *GADL1*-overexpressing cells (GADL1) (^*^p < 0.05). Data for SH-SY5Y cells cultured in the conditioned medium (CM) from SH-SY5Y cells (5Y-CM) vs. from *GADL1*-overexpressing cells (GADL1-CM) were also compared using the Student’s t test, which revealed no significant differences with respect to changes in cell migration speed and perimeter length for cells treated with lithium (**c,d**) or CCL2 (**e,f**). Effects of *GADL1* overexpression on cell migration speed (**a**), and perimeter length (**b**) were calculated from differences between SH-SY5Y and *GADL1*-overexpressing cells without any treatment (cells). The same calculations were also done for SH-SY5Y cells cultured in 5Y-CM vs. GADL1-CM (CM). The Student’s t test was used to compare the differences calculated from cells vs. CM (^***^p < 0.001).

Independent experiments were carried out to confirm our observed effects of *GADL1* overexpression or treatment with lithium or CCL2 on cell migration speed (Fig. 6a,c,e, respectively), cell perimeter length recorded at 42 h (Fig. 6b,d,f, respectively), cell irregularity recorded at 42 h (Fig. S4a,c,e, respectively), and cell eccentricity recorded at 42 h (Fig. S4b,d,f, respectively). Effects of *GADL1* overexpression on cell migration speed (Fig. 6a) were calculated from differences between SH-SY5Y and *GADL1*-overexpressing cells without any treatment (−5.2 ± 1.46 μm/h) or from differences of SH-SY5Y cells cultured in 5Y-CM vs. GADL1-CM (−0.9 ± 0.47 μm/h). Effects of *GADL1* overexpression on cell perimeter length (Fig. 6b) were calculated from differences between SH-SY5Y and *GADL1*-overexpressing cells without any treatment (−13.1 ± 4.52 μm) or from differences of SH-SY5Y cells cultured in 5Y-CM vs. GADL1-CM (−1.3 ± 1.46 μm). Effects of *GADL1* overexpression on cell irregularity (Fig. S4a) and eccentricity (Fig. S4b) were calculated from differences between SH-SY5Y and *GADL1*-overexpressing cells without any treatment or from differences of SH-SY5Y cells cultured in 5Y-CM vs. GADL1-CM, showing that only cell irregularity was affected by GADL1 overexpression. These results suggested that the *GADL1* overexpression–induced decrease in cell migration speed (Fig. 6a), perimeter length (Fig. 6b), and irregularity (Fig. S4a) were cell autonomous, depending mainly on the intracellular form of GADL1. Secreted GADL1 might have mild effects on the decrease in cell migration speed (Fig. 6a).

Lithium induced a decrease in cell migration speed (Fig. 6c), perimeter length (Fig. 6d), irregularity (Fig. S4c), and eccentricity (Fig. S4d) of SH-SY5Y and *GADL1*-overexpressing cells. However, *GADL1*-overexpressing cells were less sensitive to lithium than were SH-SY5Y cells, as reflected by the degree of decrease in migration speed (−1.5 ± 0.69 μm/h, −3.2 ± 0.15 μm/h, respectively, Fig. 6c) and perimeter length (−6.7 ± 2.14 μm, −11.9 ± 3.59 μm, respectively, Fig. 6d). The differential sensitivity to lithium was not observed when SH-SY5Y cells were cultured in the 5Y-CM vs. GADL1-CM, indicating that the differential sensitivity toward lithium depended mainly on the intracellular form of GADL1.

In comparison, CCL2 (50 ng/ml) induced an increase in cell migration speed (Fig. 6e), perimeter length (Fig. 6f), irregularity (Fig. S4e), and eccentricity (Fig. S4f) for both SH-SY5Y and *GADL1*-overexpressing cells. In addition, *GADL1*-overexpressing cells were more sensitive than SH-SY5Y cells to CCL2 treatment, especially with respect to changes in perimeter length (4.8 ± 3.77 μm, −0.9 ± 3.28 μm, respectively, Fig. 6f). The differential sensitivity to CCL2 was not evident when SH-SY5Y cells were cultured in the 5Y-CM vs. GADL1-CM, indicating that the differential sensitivity to CCL2 exposure depended mainly on the intracellular form of GADL1.

## Discussion

*GADL1* overexpression downregulated *FN1*, *ITGA2*, and *ITGAV*, which involved in cell adhesion and migration processes as well as maintenance of cell shape^22^. In our study, *GADL1* overexpression suppressed cell migration, area, volume, perimeter length, irregularity and eccentricity. The RNA expressions of *FN1*, *ITGA2*, *ITGAV*, and *CCL2* were upregulated after *GADL1* knockdown in the overexpression cells, suggesting that the observed cellular phenotype and migration changes upon *GADL1* overexpression were indeed triggered by *GADL1* overexpression itself. Cell migration speed and perimeter length exhibited similar trends, and both of them were decreased under *GADL1* overexpression or lithium treatment but increased upon stimulation with CCL2. Several reports show that cancer cells with the longer perimeter length or a greater value of irregularity can move faster, and thus resulting in the enhanced ability for invasion and metastasis^27–29^. The relationship between cell shape and migration in cancer cells is similar to our observations. For both SH-SY5Y and *GADL1*-overexpressing cells, lithium treatment decreased cell migration and perimeter length. Lithium has been reported to inhibit invasion and migration of glioma cells^30^. Lithium can regulate a cytoskeletal modulator^31^ and may also play a role in the neuron migration.

CCL2 has been found to regulate neuron migration^24,25^ and can stimulate embryonic hypothalamic neurons to migrate greater distances^25^. For both SH-SY5Y and *GADL1*-overexpressing cells, stimulation with CCL2 also increased cell number and migration speed but decreased cell thickness. In comparison, CCL2 exposure increased cell area, perimeter length, and irregularity only in *GADL1*-overexpressing cells, whereas it decreased cell volume only in SH-SY5Y cells. These results indicate that *GADL1* overexpression increased cell sensitivity to CCL2 treatment. The findings were echoed with the observation that *GADL1* overexpression downregulated *CCL2* expression.

It has been reported that taurine retards radial migration of neurons in the developing mouse cerebral cortex^32^. However, we found that *GADL1* overexpression affected cell migration, perimeter length, and the differential sensitivity to lithium and CCL2, all of which were dependent mainly on the intracellular form of GADL1. Effects of *GADL1* overexpression on cell migration speed or perimeter length were much more obvious in the differences between SH-SY5Y and *GADL1*-overexpressing cells than in the differences of SH-SY5Y cells cultured in 5Y-CM vs. GADL1-CM. These results suggested that secreted GADL1 or its enzyme product, taurine, in the conditioned medium might exert only mild effects on the observed changes.

A previous study using 3-week-old mice shows that *Gadl1* expression is higher in the olfactory bulb^18^, which is an active zone for neuron regeneration in the adult mammalian forebrain. Neuroblasts migrate tangentially along the rostral migratory stream until they reach the olfactory bulb, where they then migrate radially to complete their differentiation into neurons^19–21^. The inability of newly generated neurons in the brain to migrate to their target locations might result in improper neural circuitry maintenance and function, and thus might contribute to the emergence of neuropsychiatric disorders, including epilepsy, schizophrenia and autism^33–36^. Schizophrenia patient-derived cells are less adhesive and more mobile than cells derived from healthy control subjects^37^. Disrupted in schizophrenia1 (DISC1) regulates neuron migration, and loss of function of DISC1 may lead to schizophrenia^33^. Postmortem brains from psychiatric patients show alterations in the polysialylated neural cell adhesion molecule, a protein which has a key role in cell migration^38,39^. The level of *GADL1* expression could affect cell migration, indicating that GADL1 might play an important role in the disease development of bipolar disorder.

Exposure to 20 mM lithium decreased the number of both SH-SY5Y and *GADL1*-overexpressing cells, while treatment with lithium at 1 mM did not have powerful effects on cellular phenotypes for both cells, except on cell area and perimeter length. The possibility that lithium at the concentration of 20 mM is somewhat toxic and may have pleiotropic effects on many biological processes including cell migration could not be ruled out. To correlate the results of this experiment to clinical studies, dose-response analyses, examining the effects of lower concentrations of lithium, would be necessary in the future studies.

We found that *GADL1* overexpression decreased cell sensitivity to lithium, as reflected by its effects on cell migration speed. It has been reported that neurons transdifferentiated from bipolar patient skin cells displayed a significantly different cell-adhesion phenotype between lithium responders and nonresponders^26^. These findings together indicate that cell adhesion or migration might serve as a good indicator for the sensitivity to lithium treatment. As compared with the time-consuming and labor-intensive processes to induce neurons from patient skin cells^26^, our *GADL1* stable overexpression cell line might therefore be used as a fast screening platform for novel therapeutics for bipolar disorder. It allows high throughput chemical or RNAi-based screens of potential drug candidates for bipolar disorder. The extent of its utility will be clarified by measurement of cell-migration phenotype across different treatments; at minimum, it should facilitate efforts to elucidate mechanism of action of the gold-standard treatment for psychiatric disease.

## Methods

### Cell culture and establishment of stable cell lines

SH-SY5Y cells, a human neuroblastoma cell line, were grown in Dulbecco’s Modified Eagle Medium (DMEM)/F12 medium (1:1) (Life Technologies, USA) supplemented with 10% fetal bovine serum (FBS) (Life Technologies), 2 mM d-glutamine, 100 U/ml penicillin, and 100 μg/ml streptomycin (Life Technologies) at 37 °C in a humidified incubator containing 5% CO_2_. Full-length *GADL1* was cloned into a vector bearing an enhanced GFP reporter and the *neo* gene from Tn5 encoding an aminoglycoside 3′-phosphotransferase, which conferred resistance to G418. The plasmid with *GADL1* was transfected into SH-SY5Y cells using Xfect^TM^ transfection reagent (Clontech, USA). At 48 h post-transfection, enhanced GFP–positive cells were sorted and pooled using a FACS Aria II cell sorter (BD Biosciences, USA). The establishment of stable cell lines was achieved by selection with G418 at 300 μg/ml for 2 weeks, after which cells were maintained with 100 μg/ml G418.

### RNA expression array

Total RNA was extracted SH-SY5Y or *GADL1*-overexpressing cells pooling from sextuplicate wells using the NucleoSpin RNA/protein isolation kit (MACHEREY-NAGEL, Germany). RNA from each cell line was subjected to single microarray chip analysis. Total RNA (10 µg) was used for cDNA synthesis, and cDNA was labeled via *in vitro* transcription followed by fragmentation according to the GeneChip Expression Analysis Technical Manual rev5 (Affymetrix, USA). Labeled samples (11 µg each) were hybridized to GeneChip Human Transcriptome Array 2.0 (HTA 2.0; Affymetrix) at 45 °C for 16.5 h. The wash and staining steps were performed with a Fluidic Station-450, and the GeneChip HTA 2.0 was scanned with an Affymetrix GeneChip Scanner 7 G. Gene expression changes for the *GADL1*-overexpressing cells vs. SH-SY5Y cells were analyzed with GeneSpring software (Agilent, USA) and Ingenuity Pathway Analysis (IPA). Fold changes in gene expression were depicted using Prism 5 software (GraphPad, USA).

### Extraction of mRNA from cells and real-time quantitative PCR (RT-qPCR)

Total RNA was extracted SH-SY5Y or *GADL1*-overexpressing cells pooling from sextuplicate wells using the NucleoSpin RNA/protein isolation kit (MACHEREY-NAGEL, Germany). The extracted RNA was reverse transcribed into cDNA using a reverse transcription kit (Roche, Switzerland). Expressions of *GADL1* (PPH22451A), *FN1* (PPH00143B), *ITGA2* (PPH00625F), *ITGAV* (PPH00628C), and *CCL2* (PPH00192F) were examined with SYBR Green (Qiagen, Germany) using gene-specific primers (all designed by Qiagen) in triplicates. The primers and probe (Roche) for *ACTB* were used for the relative quantification of transcription. RT-qPCR was performed with an ABI 7500 system (Applied Biosystems, USA).

### siRNA knockdown in the *GADL1*-overexpressing cell line

24 hr after cell seeding, *GADL1*-overexpressing cells were transfected with RISC-free negative control siRNA or siRNA targeting GADL1 at 0.1 μM using DharmaFECT1 transfection reagent. Medium was changed 24 hr after transfection. Two days post-transfection, cells from sextuplicate wells were harvested and pooled for subsequent RNA extraction and reverse transcription, followed by RT-qPCR analysis for *GADL1*, *FN1*, *ITGA2*, *ITGAV* and *CCL2*. The fold-change value for each gene was normalized to *ACTB* expression.

### Digital holographic imaging

Cell migration distance and morphological changes of cells including cell area, thickness, volume, perimeter length, irregularity, and eccentricity were measured using real-time, three-dimensional holographic imaging (HoloMonitor M4; Phase Holographic Imaging, Sweden)^40^. SH-SY5Y or *GADL1*-overexpressing cells were seeded at 3 × 10^5^ cells/well on a laminin-coated 6-well plate (Corning, USA) and maintained in DMEM/F12 (1:1) with 3% FBS inside an incubator at 37 °C in 5% CO_2_. At 4–5 h after plating cells, lithium chloride (1 or 20 mM, Sigma Aldrich, USA) or CCL2 (25 or 50 ng/ml, R&D Systems, USA) was added to the cells for 48 or 72 h, respectively. Images were acquired at 20-min intervals for 48–72 h. The images were analyzed using Hstudio software (Phase Holographic Imaging). The dose of 20 mM lithium used in the experiment was based on a previous study showing that, at this dose, lithium activated MAPK and inhibited GSK-3β in SH-SY5Y cells, with no evidence of cytotoxicity^41^. Besides, 20 mM lithium treatment resulted in an intracellular lithium concentration of 3.2 ± 0.2 mM as measured by a previous study^42^.

### Collection of conditioned medium (CM)

SH-SY5Y and *GADL1*-overexpressing cells were cultured in DMEM/F12 (1:1) with 3% FBS. After 2–3 days of culture, the resultant CMs (5Y-CM and GADL1-CM) were harvested and sieved using a 0.22 μm filter. SH-SY5Y cells seeded at 3 × 10^5^ cells/well on a laminin-coated 6-well plate (Corning) were cultured in the presence of CM vs. fresh complete medium (2:3) and subjected to 2–3 days of continuous real-time holographic imaging.

### Statistical analysis

Repeated measure ANOVA with Tukey’s multiple comparison test in GraphPad Prism was used to analyze the results from holographic imaging between different cell lines or different treatments. The Student’s t test was used to compare the results from different batches of experiments.

## Supplementary information

Supplementary Information

## Data Availability

The RNA expression array datasets generated and analyzed in this study are available from the corresponding authors on reasonable request.
