# Supplementary material for: Effects of GADL1 overexpression on cell migration and the associated morphological changes
Source: Sci Rep. 2019 Mar 28;9:5298. doi: 10.1038/s41598-019-41689-x (PMC6438977; doi:10.1038/s41598-019-41689-x)
Supplement: Supplementary file 1 — Supplementary Information [file 41598_2019_41689_MOESM1_ESM.docx]

**Effects of *GADL1* overexpression on cell migration and the associated morphological changes**

**Tai-Na Wu^1^, Chih-Ken Chen^2^, I-Chao Liu^3^, Lawrence Shih-Hsin Wu^4^* and Andrew Tai-Ann Cheng^1, 4^*******

**^1^** Institute of Biomedical Sciences, Academia Sinica, Taipei, Taiwan;

**^2^** School of Medicine, Chang-Gung University and Chang-Gung Memorial Hospital, Keelung, Taiwan;

**^3^** School of Medicine, Fu Jen Catholic University and Fu Jen Catholic University Hospital, New Taipei, Taiwan;

**^4^** Graduate Institute of Biomedical Sciences, China Medical University, Taichung, Taiwan.

***Correspondence**:

Dr. Lawrence S. H. Wu, Graduate Institute of Biomedical Sciences, China Medical University, Taichung, Taiwan

Dr. Andrew T. A. Cheng, Institute of Biomedical Sciences, Academia Sinica, 128, Section 2, Academia Road, Taipei, Taiwan, 11529

Phone: +886-4-22052121 ext. 7732; Fax: +886-4-22333641; E-mail: [lshwu@hotmail.com](mailto:lshwu@hotmail.com) (Dr. L.S.H. Wu)

Phone: +886-2-27899119; Fax: +886-2-27823047; E-mail: [bmandrew@gate.sinica.edu.tw](mailto:bmandrew@gate.sinica.edu.tw) (Dr. A.T.A. Cheng)

***Supplementary Information***


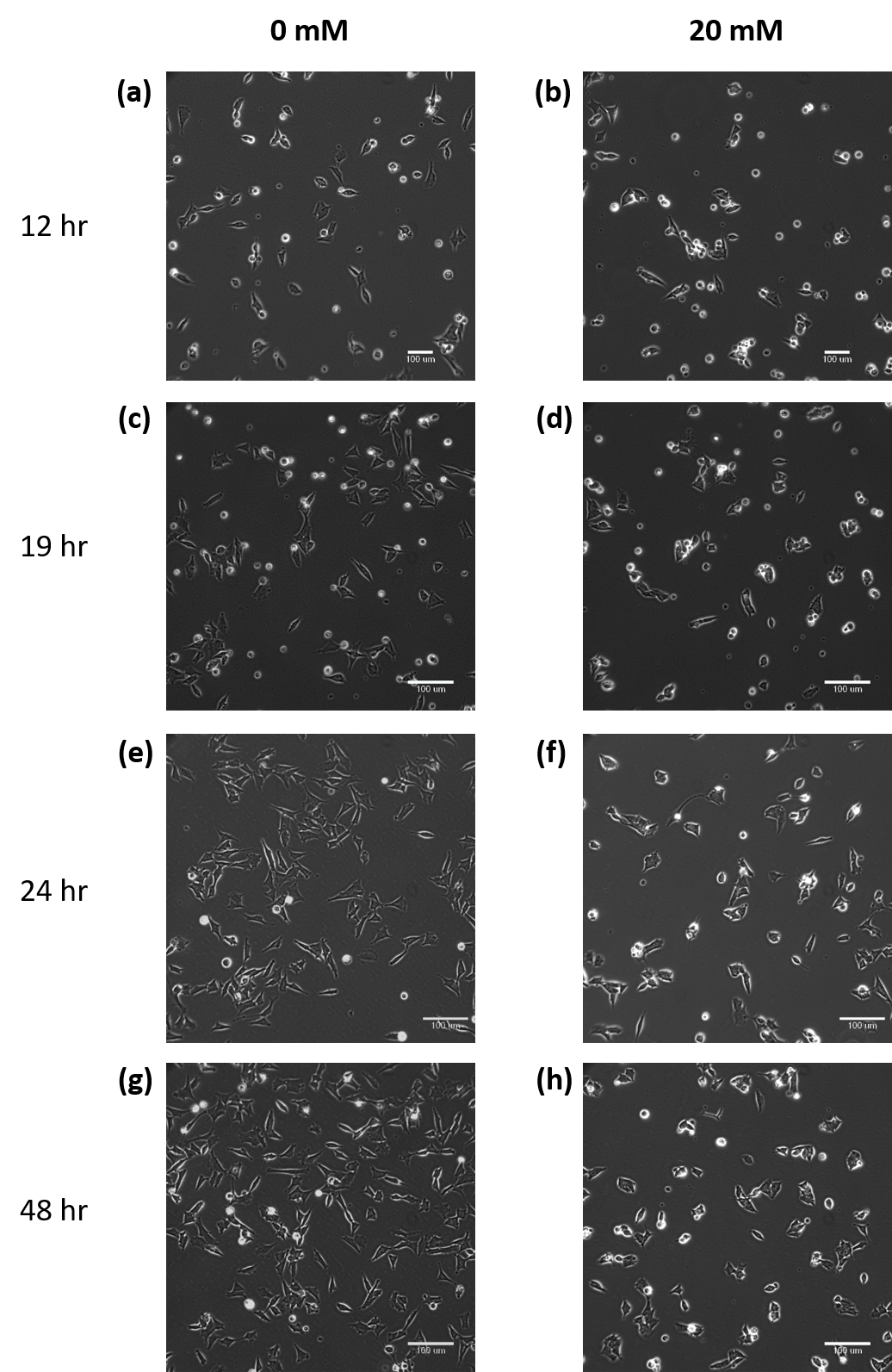


**Fig. S1 The morphological changes of SH-SY5Y cells in the presence or absence of lithium treatment.**

SH-SY5Y cells were seeded on a laminin-coated 6-well plate and maintained in DMEM/F12 (1:1) with 3% FBS inside an incubator at 37°C in 5% CO_2_. At 4–5 h after plating cells, 20 mM lithium chloride was added to the cells. Cells were recorded after 12, 19, 24 and 48 h of mock (a, c, e, g) or lithium (b, d, f, h) treatment using phase contrast microscopy.


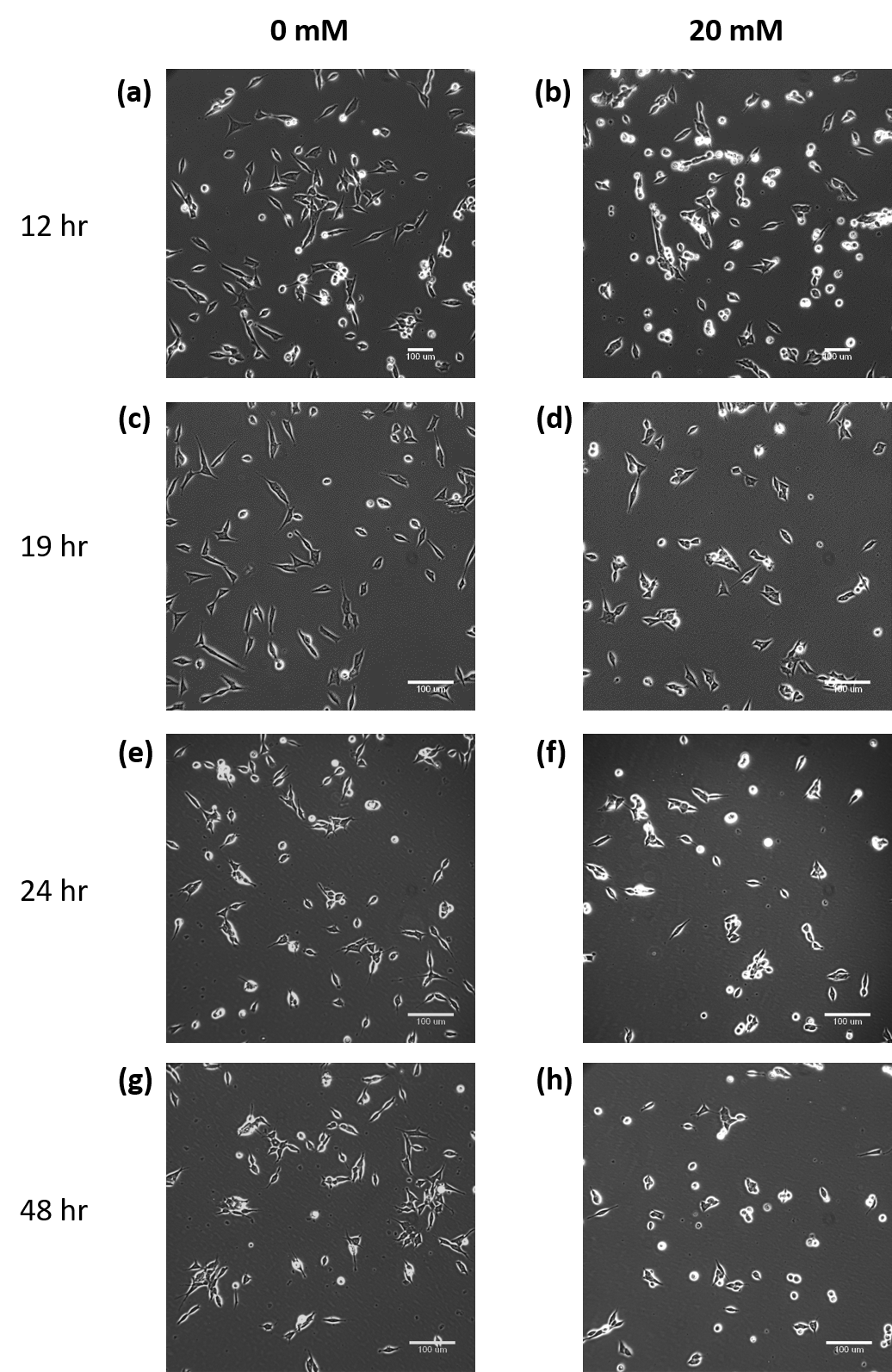


**Fig. S2 The morphological changes of *GADL1*-overexpressing cells in the presence or absence of lithium treatment.**

*GADL1*-overexpressing cells were seeded on a laminin-coated 6-well plate and maintained in DMEM/F12 (1:1) with 3% FBS inside an incubator at 37°C in 5% CO_2_. At 4–5 h after plating cells, 20 mM lithium chloride was added to the cells. Cells were recorded after 12, 19, 24 and 48 h of mock (a, c, e, g) or lithium (b, d, f, h) treatment using phase contrast microscopy.

**
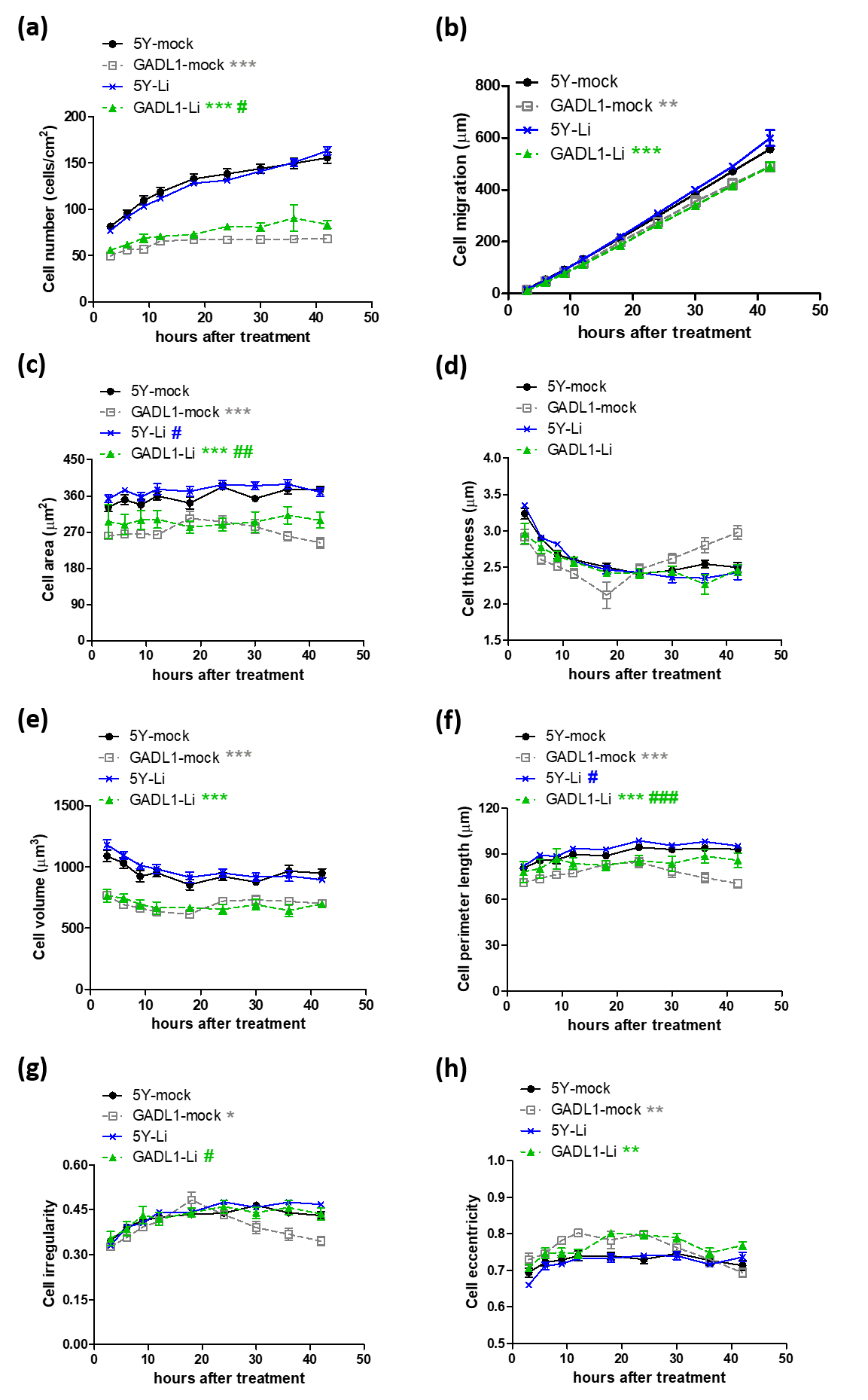
**

**Fig. S3 Low dose of lithium effects on cell number, migration and morphology.**

(a) Cell number, (b) cell migration distance, and morphological changes of cells including (c) cell area, (d) thickness, (e) volume, (f) perimeter length, (g) irregularity, and (h) eccentricity were measured using real-time, three-dimensional holographic imaging. At 4–5 h after seeding of SH-SY5Y (5Y) cells or *GADL1*-overexpressing cells (GADL1), 1 mM lithium was added, and images were acquired at 20-min intervals for 48 h. Data were mean ± s.e.m. values from one experiment and were representative of three independent experiments. Repeated measure ANOVA with Tukey’s multiple comparison test was used to compare the differences between SH-SY5Y and *GADL1*-overexpressing cells (*p < 0.05; **p < 0.01; ***p < 0.001) or between mock and lithium treatment (#p < 0.05; ##p < 0.01; ###p < 0.001).


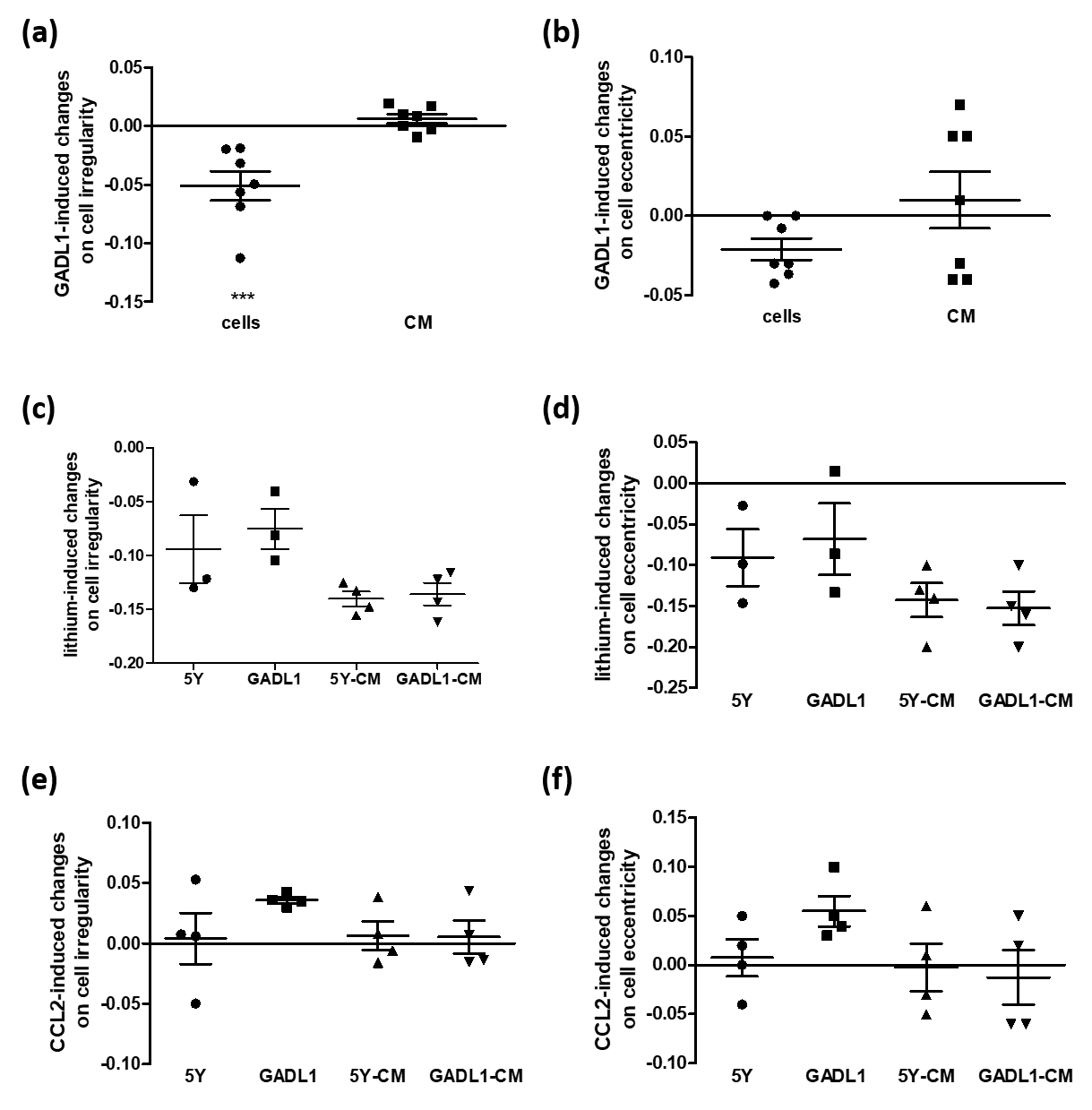


**Fig. S4 Effects of *GADL1* overexpression or lithium or CCL2 treatments on cell irregularity and eccentricity from independent experiments.**

Data from different batches of experiments were statistically analyzed to confirm *GADL1* overexpression and the effects of treatment with lithium (20 mM) or CCL2 (50 ng/ml) on cell irregularity (a, c, e, respectively), and eccentricity (b, d, f, respectively) recorded at 42 h. The Student’s t test was used to compare the differences between SH-SY5Y (5Y) cells and *GADL1*-overexpressing cells (GADL1). Data for SH-SY5Y cells cultured in the conditioned medium (CM) from SH-SY5Y cells (5Y-CM) vs. from *GADL1*-overexpressing cells (GADL1-CM) were also compared using the Student’s t test, which revealed no significant differences with respect to changes in cell irregularity and eccentricity for cells treated with lithium (c, d) or CCL2 (e, f). Effects of *GADL1* overexpression on cell irregularity (a), and eccentricity (b) were calculated from differences between SH-SY5Y and *GADL1*-overexpressing cells without any treatment (cells). The same calculations were also done for SH-SY5Y cells cultured in 5Y-CM vs. GADL1-CM (CM). The Student’s t test was used to compare the differences calculated from cells vs. CM (***p < 0.001).
